# Chemical Profile, Cytotoxic Activity and Oxidative Stress Reduction of Different *Syringa vulgaris* L. Extracts

**DOI:** 10.3390/molecules26113104

**Published:** 2021-05-22

**Authors:** Daniela Hanganu, Mihaela Niculae, Irina Ielciu, Neli-Kinga Olah, Melania Munteanu, Ramona Burtescu, Răzvan Ștefan, Loredana Olar, Emoke Pall, Sanda Andrei, Dan C. Vodnar, Daniela Benedec, Ilioara Oniga

**Affiliations:** 1 Department of Pharmacognosy, “Iuliu Haţieganu” University of Medicine and Pharmacy, 400010 Cluj-Napoca, Romania; dhanganu@umfcluj.ro (D.H.); dbenedec@umfcluj.ro (D.B.); ioniga@umfcluj.ro (I.O.); 2 Department of Clinical Sciences, University of Agricultural Sciences and Veterinary Medicine, 400374 Cluj-Napoca, Romania; mihaela.niculae@usamvcluj.ro (M.N.); loredana.olar@usamvcluj.ro (L.O.); emoke.pall@usamvcluj.ro (E.P.); 3 Department of Pharmaceutical Botany, “Iuliu Haţieganu” University of Medicine and Pharmacy, 400010 Cluj-Napoca, Romania; 4 PlantExtrakt Ltd., Rădaia, 407059 Cluj-Napoca, Romania; neli.olah@plantextrakt.ro (N.-K.O.); ramona.burtescu@plantextrakt.ro (R.B.); 5 Department of Medicinal Chemistry and Pharmaceutical Industry, Faculty of Pharmacy, “Vasile Goldiş” Western University of Arad, 310414 Arad, Romania; 6 Department of Environmental Chemistry, Faculty of Pharmacy, “Vasile Goldiş” Western University of Arad, 310414 Arad, Romania; munteanu.melania@uvvg.ro; 7 Department of Preclinic Sciences, Faculty of Veterinary Medicine, University of Agricultural Sciences and Veterinary Medicine, 400372 Cluj-Napoca, Romania; rstefan@usamvcluj.ro (R.Ș.); sandrei@usamvcluj.ro (S.A.); 8 Department of Food Science and Technology, University of Agricultural Sciences and Veterinary Medicine, 3-5 Mănăștur Street, 400372 Cluj-Napoca, Romania; dan.vodnar@usamvcluj.ro

**Keywords:** *Syringa vulgaris* L. flowers, bark, leaves, fruit, Oleaceae polyphenols, secoiridoids, antioxidant, cytotoxic

## Abstract

*Syringa vulgaris* L. (common lilac) is one of the most popular ornamental species, but also a promising not comprehensively studied source of bioactive compounds with important therapeutic potential. Our study was designed to characterize the chemical composition and to assess the antioxidant and cytotoxic properties of ethanolic extracts obtained from *S. vulgaris* L. flowers, leaves, bark, and fruit. The chemical profile of the ethanolic extracts was investigated using chromatographic (HPLC-DAD-ESI^+^, GC-MS) and spectral (UV-Vis, FT-IR) methods, while the protective effect against free radicals was evaluated in vitro by different chemical assays (DPPH, FRAP, CUPRAC). The cytotoxic activity was tested on two tumoral cell lines, HeLa, B16F10, using the MTT assay. Significant amounts of free or glycosylated chemical components belonging to various therapeutically important structural classes, such as phenyl-propanoids (syringin, acteoside, echinacoside), flavonoids (quercetin, kaempferol derivatives) and secoiridoids (secologanoside, oleuropein, **10**-hydroxy oleuropein, demethyloleuropein, syringalactone A, nuzhenide, lingstroside) were obtained for the flowers, leaves and bark extracts, respectively. Furthermore, MTT tests pointed out a significant cytotoxic potential expressed in a non-dose-dependent manner toward the tumoral lines. The performed methods underlined that *S. vulgaris* extracts, in particular belonging to flowers and leaves, represent valuable sources of compounds with antioxidant and antitumoral potential.

## 1. Introduction

The Oleaceae is an important family among flowering plants, comprising 25 genera, with over 600 species that are spread worldwide in a wide variety of habitats, especially in the temperate and subtropical climates [1,2]. The most well-known genera belonging to this family are *Olea*, *Forsythia*, *Fraxinus*, *Syringa*, *Jasminum*, and *Ligustrum*, including species that have economic importance, food and oil plants, perfumed plants, or ornamental species [2]. Among these genera, in the European flora few species can be found, especially belonging to the genera *Fraxinus* (e.g., *Fraxinus excelsior* L.), *Ligustrum* (e.g., *Ligustrum vulgare* L.), *Syringa* (e.g., *Syringa vulgaris* L.) and *Forsythia* [1]. The *Syringa* genus is one of the most widely known among these genera, being spread in the Western and Eastern part of Eurasia and comprising two species, *Syringa vulgaris* L. and *Syringa josikaea* J. Jacq. ex Rchb. The two species are differently spread along the European continent: *S. vulgaris* can be found on the Balkan Peninsula and in the southern part of the Carpathians, *S. josikaea* in the northern parts of the Carpathians and both species can be found in the western parts of the continent [3]. The genus comprises more than 40 species distributed around Europe and Asia. Most of these species are deciduous shrubs and trees [4].

The species *S. vulgaris*, the common lilac, is the most widely spread of these two species, being cultivated as an ornamental species all across the European continent [1] and for the perfumes industry [5]. In addition, the species has known various uses, especially in traditional medicine. The more frequently used medicinal product is represented by the inflorescences. In Greece, an infusion of the inflorescences is used internally to treat gastro-intestinal troubles (bloating) and externally as a massage for the treatment of gout and rheumatism [6]. Same external use is cited in the Serbian traditional medicine for this medicinal product in combination with fruit of *Aesculus hippocastanum*, for the treatment of varicose veins and rheumatism [7]. Flowers of the species are also recommended as antipyretics and appetizers as a decoction in Bulgaria, while Italian traditional medicine describes the use of bark, fruit, and leaves also as a decoction for their astringent and antipyretic effect [8]. Leaves of the species are recommended in Hungarian ethnopharmacology for the treatment of bleeding wounds, joint and muscle aches [9]. Similar species belonging to the same genus (e.g., *S. oblata* Lindl., *S. pinnatifolia* Hemsl., *S. reticulata* (Blume) H. Hara var. *amurensis* (Rupr.) J. S. Pringle, *S. pubescens* Turcz. and *S. pubescens* subsp. *patula* (Palib) M. C. Chang & X. L. Chen.) proved to have similar traditional uses. Thus, Chinese sources cite the traditional use of all these vegetal medicinal products (flowers, leaves, barks), but also of roots, branches and fruit, especially for the treatment of gastro-intestinal disorders, joint inflammations, infections, or asthma [5].

Scientific data on the species support the uses that are cited in traditional medicine, being connected to the antioxidant [10,11], antimicrobial [12], anti-inflammatory [13,14] and antipyretic properties [4,5] and inhibitory effects on blood stasis [15]. Studies were performed on similar species belonging to the genus *Syringa*, as *S. pinnatifolia* [12,16], but the vast majority were performed on the *S. vulgaris* species [1,10,11,13,14,15]. The compounds that are responsible for these activities are sesquiterpenes [12], hydroxycinnamoyl derivatives and secoiridoids or secoiridoid glycosides [1,13], phenolic compounds [10,11,14,15], lignans [14,16], phenylpropanoids and iridoids [14]. Regarding vegetal medicinal products that were tested, they were represented by flowers [10,13,15], barks [11,14,16], stems [12], fruit [10], leaves [11] or all these [1]. Nevertheless, information on this species, on its medicinal uses and on the compounds that are responsible for these uses remain scarce [1].

Taking all of this into consideration, the species of the genus *Syringa* appear to be important sources of compounds, proving at the same time important medicinal uses. These species need further investigation, in order to bring further evidence for their introduction in therapy and for the investigation of their bioactive ingredients and mechanisms of action underlying the pharmacological effects they exhibit, as these data remain scarce [1,4]. Therefore, the novelty and originality of this study is represented exactly on its main purpose, which is represented by evaluating the chemical composition and testing the antioxidant and cytotoxic activities of extracts belonging to different parts of the species: flowers, leaves, bark, and fruit. Moreover, the study aims to perform a comparison between these extracts, regarding their composition and biological activities, with the final purpose of highlighting the potential of flowers, which may represent an important medicinal product that has anti-proliferative potential and may be used in the therapy of different forms of cancer.

## 2. Results and Discussion

### 2.1. HPLC-DAD-ESI^+^ Analysis

Results obtained for the HPLC-DAD-ESI^+^ analysis of phenolic compounds can be found in Table 1.

Performed analysis clearly showed significant differences concerning the quantity of chemical compounds in the *S. vulgaris* extracts obtained from leaves, bark, fruit, and flowers. Important amounts of free or glycosylated chemical components belonging to various therapeutically important structural classes, such as phenyl-propanoids (syringin, acteoside, echinacoside), flavonoids (quercetin and kaempferol derivatives), secoiridoids (secologanoside, oleuropein, **10**-hydroxy oleuropein, demethyloleuropein, syringalactone A, nuzhenide, lingstroside) and p-coumaroyl-glycolic acid were obtained. Twelve out of 14 identified compounds were detected and quantified for in all tested extracts, while acteoside and oleuropein were not found in the fruit extract (Table 1). Furthermore, significantly high amounts of certain identified compounds, namely p-coumaroyl-glicolic acid (6748.16 μg/g), secologanoside (27,663.00 μg/g), acteoside (9408.78 μg/g), quercetin-rutinoside (7642.07 μg/g), demethyl oleuropein (35,729.89 μg/g), kaempferol-glucoside (3814.93 μg/g), nuzhenide (15,893.68 μg/g) were observed in the case of the flower extract (Table 1). Nuzhenide is a secoiridoidic compound with strong antioxidant activity, similar oleuropein and its derivatives [4,10].

The bark extract possessed the highest concentration in syringin (74,535.30 μg/g), syringalactone A (17,161.82 μg/g), **10**-Hydroxy-oleuropein (8943.89 μg/g), echinacoside (38,299.52 μg/g), ligstroside (24,820.71 μg/g), oleuropein (9139.07 μg/g), oleuropein-aglycone (42,796.39 μg/g) (Table 1). These results underlined bark extract rich content in echinacoside, a phenylpropanoid glycoside recognized for immunostimulant and antioxidant properties, and syringin, a phenylpropanoid glycoside with immunostimulatory, antioxidant and antidiabetic properties [17]. Moreover the bark syringin presence was also highlighted in the other analyzed parts of the plant (14,653.98 μg/g for flowers extract, 9245.97 μg/g for leaves extract).

Overall, flowers extract revealed the highest and most balanced content of phenylpropanoid, flavonoid and secoiridoid compounds, while the fruit extract presented the lowest content of these active compounds.

Although literature does not document detailed information regarding the chemical profile of ethanolic extracts, the polyphenolic composition was assessed in the case of extracts obtained from lilac flowers [10,15], fruit [10], bark [11,14] and leaves [11,15,18]. Tóth et al. performed the HPLC-DAD-ESI-MS analysis of methanolic extracts obtained from lilac flowers and fruit and indicated 34 compounds, including 18 secoiridoids, seven phenylpropanoids, four flavonoids and five low molecular weight phenols. Flowers were found to contain significant amounts of phenylpropanoids (acteoside, 2.48%; echinacoside, 0.75%) and oleuropein (0.95%), while the fruit’s major secondary metabolites were identified as secoiridoids oleuropein (1.09%) and nuzhenide (0.42%) [10]. Another study pointed out a complex metabolite profile and the antioxidant potential in the case of *Syringa vulgaris* bark and leaf methanolic extracts. A total of 33 compounds (15 secoiridoids, 6 phenyl-propanoids, 3 flavonoids, 3 lignans and 6 low molecular weight phenols) were identified by HPLC-DAD-ESI-TOF and HPLC-DAD-ESI-MS/MS. The main phenolic compounds in bark and leaves were represented by syringin (2.52%) and rutin (1.13%) respectively [11]. Filipek et al. reported the identification of 22 compounds: five simple phenolic compounds, four lignans, three phenylethanoids, a phenylethanoid esterified with an oleoside and eight secoiridoids and iridoids. Among these compounds, syringin, acteoside, nuzhenide, echinacoside, oleuropein, and ligstroside were the most important [14].

Taking all of this into consideration, the present study brings novelty and originality, being, to the best of our knowledge, the only one comparing the four different extracts (obtained from flowers, leaves, bark and fruit), reporting large amounts of bioactive compounds in their composition and showing therefore that flowers represent the most important source of active principles.

### 2.2. Vibrational IR Spectra of Syringa vulgaris L., Bark and Fruit Extracts

Infrared spectroscopy is an effective and non-targeted analytical method which could non-destructively and cost-effectively detect the intrinsic quality of different plants [19,20]. Based on FT-IR results, it is possible to highlight a list of peaks that can be assigned to different biochemical class of compounds, which could lead to a better correlation between the chemical structure and the spectroscopic features of plants [21]. Therefore, this technique also provides a precise assignment of the functional groups, bonding types and molecular conformations within plant tissue and cells [20].

**Figure 1:**
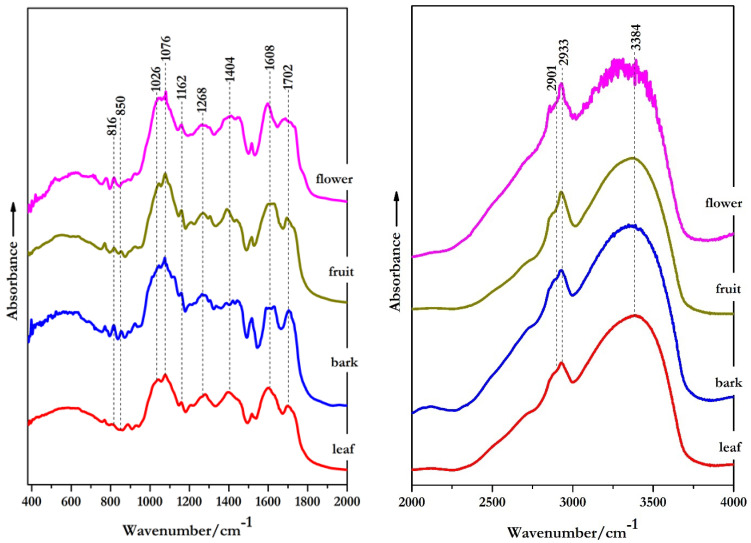
The FT-IR absorbance spectra of *S. vulgaris* flowers, bark, leaves and fruit extracts. The profiles are presented in the wavenumber range 400–2000 cm^−1^ (**left**) and 2000–4000 cm^−1^ (**right**).

The FT-IR spectra of analyzed *S. vulgaris* extracts showed some spectral changes (Figure 1). Moreover, as it can be observed in Table 1 and Table 2, the main functional groups of echinacoside (a phenyl-propanoid), rutin (a flavonoid) and iridoids were illustrated in the FT-IR spectra of *S. vulgaris* extracts. Thus, peaks between 814–816 cm^−1^, 1513–1515 cm^−1^ were characteristic to phenyl-propanoid-associated signals of echinacoside [21,22]. Within a similar range, terpene-associated stretching vibrations of C=O had contribution to band from 1694–1702 cm^−1^ [23]. Another terpene-associated peak was observed in the range 2901–2909 cm^−1^ and was caused by the C–H stretching vibration [20,23,24,25]. Furthermore, the peaks found between 885–890 cm^−1^ and 2925–2933 cm^−1^ were associated with the presence of CH_2_ functional group, while the peak between 1256–1268 cm^−1^ could be attributed to C–O stretching vibration from both terpene and iridoids [23]. A strong peak in the range 1076–1079 cm^−1^ was caused by iridoids stretching vibration of –C–OH [24,25,26]. Presence of flavonols in the *S. vulgaris* analyzed extracts could also be confirmed. Therefore, the peak occurring within range 1596–1608 cm^−1^ could be attributed to C=C stretching vibrations from the structure of both flavonols and iridoids [24,26,27,28]. Similarly, in the FT-IR spectra of *S. vulgaris* analyzed extracts, flavonols possess C–O–C, C=C and CH_3_ functional groups which display peaks between 924–930 cm^−1^, 1159–1162 cm^−1^ and 1385–1404 cm^−1^ respectively [20,23,27,28,29] (Table 2 and Table 3).

Another observation is that the bands in the region 1000–1800 cm^−1^ are of much higher intensity compared to the bands bellow 1000 cm^−1^. Additionally, in the same region one can observe that some bands are shifted to lower or higher wavenumbers.

### 2.3. Quantification of Total Polyphenolic (TPC), Flavonoids (TFC) and Phenolic Acids Content (TPA)

The total polyphenols, flavonoids, and phenolic acids content of the *S. vulgaris* tested extracts showed significant amounts of all these compounds that could be corroborated with the results obtained for the assessment of the biological activities (Table 4).

Quantification of TPC, TFC and TPA are reported hereby for the first time in scientific literature for the *S. vulgaris* species, representing a further reason that sustains the originality of this study and offering important arguments in order to support the biological activities that are tested.

### 2.4. GC-MS Analysis

Results obtained for the GC-MS analysis of different *S. vulgaris* extracts can be found in Table 5, Table 6, Table 7 and Table 8.

The GC-MS analysis allowed to identify the main volatile compounds in the *S. vulgaris* flowers extract that are the two steroisomers of the lilac alcohols, lilac alcohol C and lilac alcohol D. Together with these, other aromatic compounds, phenols and acids, fatty acids could be identified in the composition of the flowers (Table 5).

**Figure 2:**
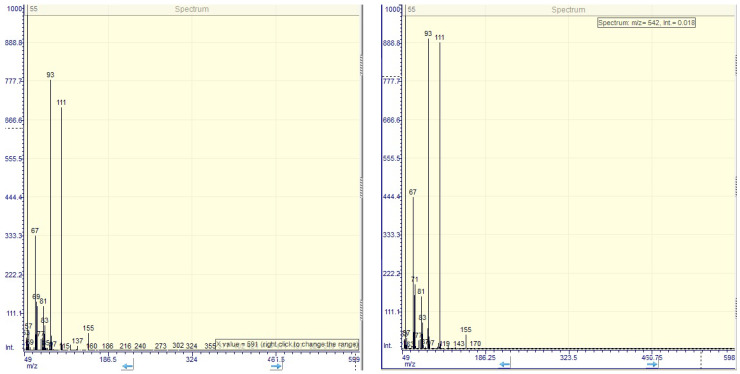
The MS spectra of lilac alcohol C (**left**) and D (**right**) separated from *Syringa vulgaris* flowers extract.

Twenty compounds were identified and among these the ones that were found in the highest amounts are the lilac alcohols and the benzyl alcohol, with matching factors higher than 80. Furane derivatives were also identified in high amounts, being related to lilac alcohols. The specific compounds, lilac alcohols, as furane alcohols, represented more than 40% of the identified compounds. The two compounds are two different stereoisomers with similar MS spectra (Figure 2) that corresponds with those of standard data from PubChem. They have a major signal at *m*/*z* of 55 and other important signals at *m*/*z* of 67, 93 and 111.

Together with lilac alcohols, another important compound that is responsible for the aromatic smell of lilac flowers is the benzyl alcohol, found in high amount 1.77% of the identified compounds are furane derivatives, compounds that are related to lilac alcohols.

Another compound that was identified in a significant amount is methyleugenol, a phenypropanoidic compound with important antioxidant activity. The phenolic compounds percentage is at 1.74% and they are being represented, moreover methyleugenol, by the ester of p-hydroxycinnamic acid fatty acid esters and free fatty acids were also identified that represents 4.52% of the identified compounds.

For the other tested extracts, GC-MS analysis was performed in the same conditions and different compounds were identified (Table 6, Table 7 and Table 8). The common compound identified in all these extracts is the n-hexadecanoic acid, a saturated fatty acid, named also palmitic acid. The fruit extract is rich in fatty acids, the main being the trans-13-octadecanoic acid. The leaves extract contains benzoic acid derivatives and also phellandrene. The bark extract contains carotenes: astaxanthin and psi-carotene respectively more than 12% trans-sinapyl alcohol.

The GC-MS analysis of these extracts is reported hereby for the first time, representing an important tool for the identification of the main compounds found in the composition of different extracts obtained from different parts of the species that are responsible for the biological activities.

### 2.5. Antioxidant Activity Assays

The in vitro antioxidant capacity of *S. vulgaris* extracts was evaluated by three different methods: 2,2-diphenyl-picrylhydrazil (DPPH●) scavenging assay, ferric-reducing antioxidant power (FRAP) and cupric ion reducing antioxidant capacity (CUPRAC) (Table 9). The three used methods were chosen in order to bring arguments that prove the antioxidant capacity of tested samples by three different mechanisms [31,32,33].

Antioxidant activity for the flowers and fruit of *S. vulgaris* are reported by Tóth et al., using the DPPH bleaching assay and revealed an effective antioxidant activity of these methanolic extracts with IC_50_ = 65.25 µg/mL and IC_50_ = 67.39 µg/mL, respectively [10]. The same assay is used by Varga et al. and showed a superior antioxidant activity of leaves and bark extracts (IC_50_ = 25.25 µg/mL, IC_50_ = 40.61 µg/mL respectively) [11]. Our study revealed that the flowers extract showed significant (*p* < 0.001) higher antioxidant activity (IC_50_ = 36.83 µg/mL) compared to the other analyzed extracts in the following order: flowers > leaves > bark > fruit (Table 9). The similar results for flowers extract were obtained by CUPRAC and FRAP assays (*p* < 0.001). Moreover, the present study brings originality by offering, for the first time, proof of antioxidant capacity of the tested samples by different other mechanisms that are highlighted using the two other assays, FRAP and CUPRAC. At the same time, the study offers for the first time a comparative view on flowers, fruit, leaves and bark, bringing evidence on the fact that flowers represent the most important antioxidant potential.

Moreover the originality that is brought by the performed assays, the study of the antioxidant capacity of the tested samples becomes even more important, as it represents the basis of the cytotoxic activity. The antioxidant capacity is highly related to the phenolic composition of the tested samples [10,11], as it is largely known and accepted that phenylpropanoids, flavonoids and also secoiridoids exhibit antioxidant and antiproliferative capacity [34,35,36], but moreover, the antioxidant capacity can be, at the same time, highly related to the cytotoxic activity of these samples, as the antioxidant activity may represent one of the most important mechanisms at the basis of the cytotoxicity [37].

### 2.6. Cytotoxiciy Assays

**Figure 3:**
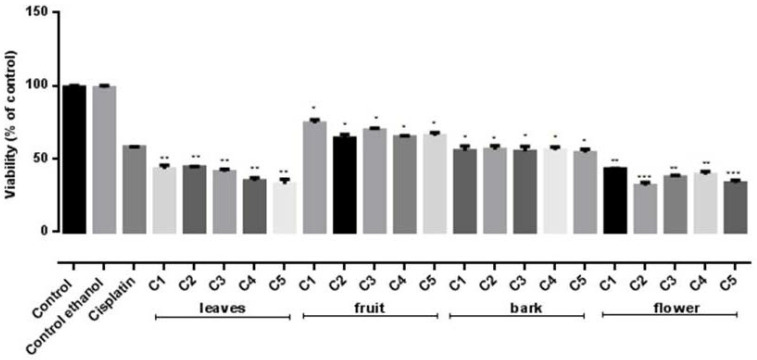
Inhibitory effects on Hela cell line of *S. vulgaris* leaves, fruit, bark and flowers extracts at five different concentrations C1–C5 calculated according to the TPC (µmol GAE/mL) determined for each extracts: leaves (11.34–56.7 µmol GAE/mL), fruit (6.66–33.2 µmol GAE/mL), bark (9.875–49.37 µmol GAE/mL) and flowers (11.69–58.475 µmol GAE/mL); Negative control—untreated cells, Internal control—Ethanol, Positive control—Cisplatin. Values represent the mean ± SD of three determinations. * *p* < 0.05; ** *p* < 0.001; *** *p* < 0.0001 (Differences between extract—treated cells and the negative control).

**Figure 4:**
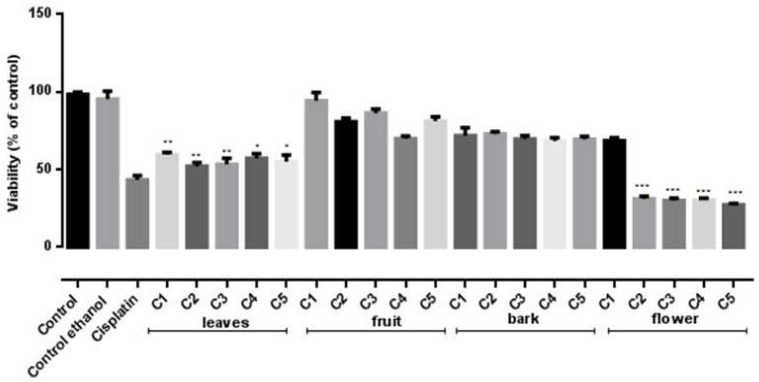
Inhibitory effects on B16H10 cell line of *S. vulgaris* leaves, fruit, bark and flowers extracts at five different concentrations C1–C5 calculated according to the TPC (µmol GAE/mL) determined for each extracts: leaves (11.34–56.7 µmol GAE/mL), fruit (6.66–33.2 µmol GAE/mL), bark (9.875–49.37 µmol GAE/mL) and flowers (11.69–58.475 µmol GAE/mL); Negative control—untreated cells, Internal control—Ethanol, Positive control—Cisplatin. Values represent the mean ± SD of three determinations. * *p* < 0.05; ** *p* < 0.001; *** *p* < 0.0001 (Differences between extract—treated cells and the negative control).

To investigate the in vitro antiproliferative potential of the four *S. vulgaris* ethanolic extracts on two human cancer cell lines, HeLa and B16F10, the MTT assay was conducted. Results are presented in Figure 3 and Figure 4.

The performed analysis indicated a relevant in vitro cytotoxic activity for all tested extracts and differences in regard to the tested doses and cancer cell lines. The most intense in vitro cytotoxicity was noticed in the case of the flowers, followed by leaves and bark extracts, respectively.

The flowers ethanolic extract displayed significant cytotoxic activity on both B16F10 and HeLa cells (Figure 3 and Figure 4). The viability of both melanoma and carcinoma cells was significantly decreased (*p* < 0.0001) compared to the untreated control for concentrations of 23.39–58.475 µmol GAE/mL and 11.69–58.475 µmol GAE/mL, respectively. No statistically significant (*p* > 0.05) differences between the viability values determined by these concentrations were noticed, indicating that the cytotoxic activity is not dose dependent. Similar to the flowers extract, the leaves extract showed significant cytotoxic effect on both cell lines (*p* < 0.001). Furthermore, when tested against HeLa cell line, these two extracts’ inhibitory activity was comparable to the positive control—Cisplatin treated cells (Figure 3). On the B16F10 cells, a similar efficacy was displayed only by the flower extract, while the leaf and bark extracts possessed a lower cytotoxic effect compared to Cisplatin. The fruit extract proved the lowest cytotoxic effect (Figure 4).

Thus, our results demonstrate the in vitro cytotoxic properties of *S. vulgaris* extracts on two tumoral cell lines. HeLa cell line proved to be more susceptible than B16F10 cells; varying in vitro and in vivo sensitivity between differing tumor cell types is documented by literature [38,39].

A non-linear regression analysis of the dose–response curve determined half maximal inhibitory concentration (IC_50_) values for cytotoxic activity and the results are described in Table 10.

These results indicate that the lowest IC_50_ values in the case of *S. vulgaris* ethanolic extracts were obtained for fruit and bark, respectively. However, these values are calculated according to the TPC (µmol GAE/mL) determined for each extract. Thus, considering the complex chemical composition established for all four extracts, correlations between the in vitro antiproliferative activity and identified groups and/compounds were determined. No correlation was found between cell viability with TPC (r^2^ = −0.4). A strong correlation was noticed between the inhibitory effect on cell viability and certain identified and quantified compounds that are described in Table 1. The highest quantity of the following compounds **1**, **2**, **5**, **7**, **9**, **11**, **13** and **14** was attributed to the flowers extract chemical composition, while the rest of the compounds were the most aboundant in the bark extract (Table 1). Among these compounds, acteoside and echinacoside quantities appeared to strongly correlate with the cytotoxicity displayed by both flower (r^2^ = 0.84–0.97 and 0.87–0.99, respectively) and bark (r^2^ = 0.87–0.94 and 0.85–0.99, respectively) extracts toward B16H10 cell line. In the case of HeLa cell line, the correlation was observed for ligstroside (r^2^ = 0.89–0.99), syringalactone A (r^2^ = 0.90–0.99) and oleuropein-aglycone (r^2^ = 0.95–0.98). Although all these three secoiridoids compounds were found at the highest quantity in the case of the bark extract, correlation coefficients between their contents in the bark extracts and antiproliferative activity were relatively low (r^2^ = −0.80–0.49).

These findings underline phenyl-propanoids (acteoside and echinacoside) and secoiridoids (ligstroside, syringalactone A, oleuropein-aglycone) among the compounds responsible for the *S. vulgaris* ethanolic flower extract antiproliferative efficacy toward B16H10 and HeLa cell line, respectively. These results are in agreement with previous reports demonstrating in vitro antiproliferative efficacy of secoiridoids [4].

Previous studies pointed out antioxidant and antitumor potential in the case of several *Syringa* species, suggesting these properties might be related to certain major identified compounds, namely iridoids and lignans [4,5]. Aqueous extracts obtained from flowers and leaves of *S. pubescens* were in vitro cytotoxic when tested against L2215 cell line, while *S. patula* floral buds extract and two isolated compounds, syringaresinol and oleoside 11-methyl ester, were able to inhibit HepG2 cells proliferation [4,5]. Similarly, oleuropein and 3, 4-dihydroxyphenylethyl alcohol 8-*O*-β-d-glucopyranoside, two compounds isolated from *S. pubescens* subsp. *patula*, expressed cytotoxicity against P-388, L-1210, SNU-5 and HL-60 cells [5].

Several compounds isolated from *S. vulgaris* leaves were reported to exhibit antitumoral potential [4,40]. Relatively weak cytotoxicity was highlighted in the case of isooleoacteoside and syringopicroside B when tested against LOX-IMVI melanoma cell line and NCI-H522 lung cancer cell line, respectively [4,18]. Additionally, the hydrolysis product of isooleuropein, displayed moderate cytotoxic activity against lung cancer cell lines DMS273 and DMS114 [4,40].

With all these in view and corroborating the results obtained in the phytochemical analysis, in the citotoxicity assays and in the antioxidant assays, the link between all these becomes obvious and it can be concluded that the antiproliferative activity of the tested samples is significant and may be due to an antioxidant mechanism. To the best of our knowledge, this is the first study aimed to evaluate and compare the chemical profile, antioxidative and antiproliferative properties of ethanolic extracts obtained from flowers, leaves, bark, and fruit of *S. vulgaris*.

## 3. Materials and Methods

### 3.1. Chemicals and Reagents

Acetonitrile for the HPLC-DAD-MS analysis was purchased by Merck (Darmstadt, Germany), while water was purified with a Direct-Q UV system by Millipore (Darmstadt, Germany). Chlorogenic acid, rutin and oleuropein (analytical purity) were purchased from Sigma Aldrich (Darmstadt, Germany). All other chemicals used were purchased from Alfa-Aesar, Karlsruhe, Germany. The cytotoxicity was tested on two tumoral cell lines: murine melanoma cells (B16F10 cells) and human cancer cell line (HeLa). The selected cell lines were obtained from American Type Cell Collection (ATCC) (Manassas, VA, USA). The B16F10 cells were maintained in RPMI 1640 medium (Sigma Aldrich, Darmstadt, Germany) supplemented with 10% fetal bovine serum (EuroClone, MI, Pero, Italy) and 1% Antibiotic-Antimycotic 100× (Sigma Aldrich, Darmstadt, Germany). Hela cells were maintained in DMEM/F-12 medium (Gibco Life Technologies, Paisley, UK) supplemented with 10% fetal bovine serum (EuroClone, MI, Pero, Italy) and 1% Antibiotic-Antimycotic 100× (Sigma Aldrich, Darmstadt, Germany). The two cell lines were cultured in a 5% CO_2_ incubator (Advantage-Lab, Schilde, Belgium) at 37 °C in a humidified atmosphere. Cisplatin (Ebewe Pharma Ges.m.b. H. Nfg. KG, Unterach am Attersee, Austria) was included as standard positive control for cytotoxicity assay.

### 3.2. Plant Material and Preparation of Extracts

The vegetal material was harvested from Cluj county, North Western Romania. Flowers and leaves were harvested in April–May 2020, during flowering period of the species, while fruit and bark were harvested in September 2020. Voucher specimens for the harvested species are deposited in the herbarium of the Pharmacognosy Department of the Faculty of Pharmacy Cluj-Napoca (Voucher no83).

The harvested samples were air dried. For the obtention of extracts, grinded vegetal material of each sample were cold macerated with 70% *v*/*v* ethanol, in a ratio of 1:10. The solution was then subjected to percolation, filtered and used for the phytochemical analysis and biological activity testing of antioxidant capacity and cytotoxic activity [41].

### 3.3. HPLC-DAD-ESI^+^ Analysis of Polyphenolic Compounds

Evaluation of polyphenolic compounds was performed on a HP-1200 liquid chromatograph, which was equipped with a quaternary pump, autosampler, DAD detector and MS-6110 single quadrupole API-electrospray detector (Agilent-Techonologies, Santa Clara, CA, USA). Detection of phenolic compounds was carried out in positive ionization mode. Different fragmentor, in the range 50–100 V, were applied. Separation of compounds was performed on a Eclipse XDB-C18 (5 μm; 4.5 × 150 mm i.d.) column (Agilent), using as a mobile phase 0.1% acetic acid in water (A) and 0.1% acetic acid in acetonitrile (B). a multistep linear gradient was employed for elution, with the following composition: 5% B for 2 min; from 5% to 90% of B in 20 min, hold for 4 min at 90% B, then 6 min to arrive at 5% B. Flow rate was maintained at 0.5 mL/min and temperature at 25 ± 0.5 °C. The phenolic compounds in the extract were analyzed by comparing the retentions times, UV visible and mass spectra of each separated compound with three reference standards, as follows: for the flavonoids, compounds were quantified using the calibration curve of rutin obtained using five different concentrations, varying from 10 to 80 μg/mL and expressed as equivalents of rutin (mg rutin/g plant material (R^2^ = 0.9973)), for phenyl-propanoids, the compounds were quantified using the calibration curve of chlorogenic acid obtained using five different concentrations, varying from 10 to 50 μg/mL and expressed as equivalents of chlorogenic acid/g plant material (R^2^ = 0.9937), while for iridoids the compounds were quantified using the calibration curve of oleuropein obtained using five different concentrations, varying from 10 to 100 μg/mL and expressed as equivalents of chlorogenic acid/g plant material (R^2^ = 0.9966). Positively charged ions were detected by mass spectrometry, using the Scan mode. The following conditions for mass spectrometry were used: gas temperature 3500 C, nitrogen flow 7 L/min, nebulizer pressure 35 psi, capillary voltage 3000 V, fragmentor 100 V and *m*/*z* 120–1200. Chromatograms were recorded at λ = 280 and 340 nm. Data acquisition was performed using the Agilent ChemStation software [42,43].

### 3.4. FT-IR Spectroscopy

The Fourier Infrared transform spectroscopy was made at the Spectroscopy laboratory from Life Sciences Institute “King Michael I of Romania” from Cluj-Napoca. Prior to FT-IR analysis, the *S. vulgaris* leaf, flower, bark and fruit extracts were dried on a clean microscope slide for 12 h at room temperature. The dried extracts were then removed from the microscope slide and mixed with the KBr powder in a proportion of 1:100 and placed in spectral pellet press chamber steel kit. To form the translucent KBr pellet we applied a pressure of 10 t for 2 min. The FT-IR spectra were collected with a Jasco FT-IR 4100 spectrometer (Jasco, Germany), in the 4000–400 cm^1^ spectral range, using 256 scans/sample at 4 cm^−1^ resolution. Furthermore, the obtained Ft-IR spectra were corrected for CO_2_ and H_2_O using the Spectra Manager program of the same used software. Finally, the FT-IR data analysis was carried out using OriginPro Version 8.5.1 software (OriginLab Corporation, Northampton, MA, USA).

### 3.5. Quantification of Total Polyphenols, Flavonoids and Phenolic Acids Content

Total phenolic content (TPC) was assessed by a spectrophotometric method based on the color reaction of polyphenols with the Folin-Ciocâlteu reagent, according to the European Pharmacopoeia, using a calibration curve of gallic acid (R^2^ = 0.9928). Results were expressed as mg gallic acid equivalents (GAE)/g dried vegetal material. Determination of total flavonoids (TFC) was also performed by a spectrophotometric, using the aluminum chloride method, based on a calibration curve of rutin (R^2^ = 0.9981) and expressing results as mg of rutoside equivalents (RE)/g dried vegetal material. Total phenolic acids (TPA) was assessed by a spectrophotometrical method, using Arnow’s reagent, similar to the one existing in the 10th Edition of the Romanian Pharmacopoeia (Cynarae folium monograph). Results of the TPA determination were expressed as mg caffeic acid equivalents (CAE)/g dried vegetal material and calculated using a caffeic acid calibration curve graph (R^2^ = 0.9956). All these experiments were performed in triplicate [31,33,44].

### 3.6. GC-MS Analysis

The GC-MS analysis was carried out on a Dani Master GC-MS System. A SH-Rxi-5 ms column with 30 m × 0.25 mm × 0.25 µm was used for the separation of compounds. Nitrogen was used as carrier gas, with 10 mL/min flow rate. The temperature of the system followed the gradient found in Table 11.

Five µL of each sample, diluted 1 to 10 with absolute ethanol, were injected. The EIS-MS detector identified compounds with molecular weight from 50 to 600 daltons. The ion source was operated at 200 °C. The compounds were tentatively identified based on the matching factor, using the NIST MS 2.2 spectra database. A matching score higher than 80% was considered acceptable for identification of the compounds. Quantitative analysis was performed by area normalization method and the results were express as area percentage (%) [31,45].

### 3.7. Antioxidant Activity Assays

#### 3.7.1. DPPH Radical Scavenging Activity

For assessing the antioxidant capacity of *S. vulgaris* extracts, the DPPH bleaching assay was used. It is a spectrophotometric method, based on the reaction of the DPPH● reagent and antioxidants that are present in tested extracts mL of each extract of different concentrations were added to 2 mL 0.1 g/L DPPH● methanolic solution and maintained at 40 °C in a thermostatized bath, for half an hour. Absorbance and their variation were measured at 517 nm. Inhibition of the DPPH● radical was calculated using the formula: DPPH scavenging ability% = (A_control_ − A_sample_/A_control_) × 100, where A_control_ is the absorbance of control, composed of the DPPH● radical solution + methanol (a mixture containing all reagents except the tincture) and A_sample_ is the absorbance of DPPH radical + samples. Percentage of DPPH decrease was expressed in Trolox equivalents (TE, R^2^ = 0.987). DPPH radical scavenging activity of the tincture was expressed as IC_50_ (µg/mL). Assays were performed in triplicate [32,33,46].

#### 3.7.2. Ferric-Reducing Antioxidant Power Assay (FRAP)

The FRAP method is a spectrophotometric method based on the color change of a 2,4,6-tri(2-pyridyl)-1,3,5-triazine (TPTZ) radical complex with Fe^3+^. This color change is assessed by the reduction of the ferric ion (Fe^3+^) to the ferrous ion (Fe^2+^) in this complex [47]. The FRAP reagent consists of a mixture of 2.5 mL of a 10 mM TPTZ solution in 40 mM HCl, mixed with 2.5 mL 20 mM ferric chloride solution and 25 mL of acetate buffer at pH = 3.6. Of each tested sample, 4 mL was diluted to 1.8 mL with water and mixed with 6 mL of this reagent. Blank solution was prepared in the same manner, but replacing extracts with water. Antioxidant capacity was evaluated in correlation with the color change, by measuring absorbances at 450 nm, using Trolox as a reference and a calibration curve (R^2^ = 0.992). Results were expressed as µM Trolox equivalents/g dry weight vegetal product and the assays were performed in triplicate [48].

#### 3.7.3. Cupric Ion Reducing Antioxidant Capacity (CUPRAC)

The CUPRAC method is a spectrophotometric method based on the reduction of the copper ion (II) to the copper iron (I) in the neocupreine (2,9-dimethyl-1,10-phenantroline) complex. This reduction determines a color change from light green to red-orange. The change of color was correlated with the antioxidant capacity by measuring the absorbance at 450 nm. The calibration curve was plotted using concentrations of the Trolox standard and results were expressed as mM Trolox equivalent/g dry weight vegetal product [32,49,50].

### 3.8. Cytotoxicity Assays

Cytotoxicity study on tumoral cell lines was performed using the MTT assay (3-(4,5-dimethylthiazol-2-yl)-2,5-diphenyl tetrazolium bromide; Sigma Aldrich) [51,52]. For both cell lines, the cells were plated (1 × 10^5^ cells/well) in 96-well plates for 24 h in normal propagation media (200 µL cell suspention in each well). The extracts were added to the complete medium in five distinct volums (5 µL, 10 µL, 15 µL, 20 µL, 25 µL), with the resulting concentrations (C1, C2, C3, C4 and C5) calculated according to the TPC determined for each extracts and expressed as µmol GAE/mL as follows: flowers (C1—11.69 µmol GAE/mL, C2—23.39 µmol GAE/mL, C3—35.08 µmol GAE/mL, C4—46.78 µmol GAE/mL, C5—58.475 µmol GAE/mL), leaves (C1—11.34 µmol GAE/mL, C2—22.68 µmol GAE/mL, C3—34.02 µmol GAE/mL, C4—45.36 µmol GAE/mL, C5—56.7 µmol GAE/mL), bark (C1—9.875 µmol GAE/mL, C2—19.75 µmol GAE/mL, C3—29.625 µmol GAE/mL, C4—39.5 µmol GAE/mL, C5—49.37 µmol GAE/mL) and fruit (C1—6.66 µmol GAE/mL, C2—13.32 µmol GAE/mL, C3—19.98 µmol GAE/mL, C4—26.64 µmol GAE/mL, C5—33.2 µmol GAE/mL). The negative control was represented by cells lines cultured in normal expansion medium (untreated cells), while 70% *v*/*v* ethanol and Cisplatin (0.2 uM) were included as the internal control and the positive control, respectively.

The cells viability following 24 h incubation at 37 °C in a humidified atmosphere with 5% CO_2_ was evaluated using the MTT assay according to previously published protocol [51,52]. The formazan particles formed by adding 0.5 mg MTT to each well were dissolved with dimethyl sulfoxide (DMSO) (Sigma Aldrich, St. Louis, MO, USA), and the absorbance was read at 450 nm using a microplate reader (Bio-Rad, Hercules, CA, USA). The cell viability percentages (%) were calculated based on the absorbance ratio between cell cultures treated with extracts and the negative controls (untreated cells) multiplied by 100. For each extract, the cytotoxic activity expressed as IC_50_ values representing the extract concentration required to inhibit 50% of cell proliferation were calculated from the dose response curve obtained using non-linear regression. All experiments were performed in triplicates.

### 3.9. Statistical Analysis

All statistical analyses were conducted using ANOVA GraphPad Prism software, version 6.0 (GraphPad, San Diego, CA, USA). The results were expressed as the mean ± standard deviation (SD). One-way analysis of variance (ANOVA) was used, followed by Tukey’s post hoc test, to determine statistical significance. The Pearson correlation analysis was performed to determine the correlation between extracts cytotoxic activity, total phenolic content and identified compounds, respectively. A *p* value lower than 0.05 was considered statistically significant.

## 4. Conclusions

To the best of our knowledge, this is the first study aimed at evaluating and compare the chemical profile, antioxidative and antiproliferative properties of ethanolic extracts obtained from flowers, leaves, bark, and fruit of *S. vulgaris*. The performed methods highlighted that *S. vulgaris* extracts, in particular the ones obtained from flowers and leaves, are valuable sources of compounds with significant antioxidant and cytotoxic potential.

## Figures and Tables

**Table 1:** Quantification and identification of phenolic compounds (μg/g dry vegetal product) in different *S. vulgaris* extracts by HPLC-DAD-ESI^+^ analysis.

Peak No.	Compound	R_t_ (min)	UV λ_max_ (nm)	[M + H]^+^ (*m*/*z*)	*Syringa vulgaris* Leaves	*Syringa vulgaris* Bark	*Syringa vulgaris* Flowers	*Syringa vulgaris* Fruit
1	p-Coumaroyl-glycolic acid	3.16	332	223	3814.80 ± 0.96	788.46 ± 0.58	6748.16 ± 2.03	1094.85 ± 1.05
2	Secologanoside	9.71	233	391	17,539.42 ± 0.5	3020.98 ± 1.65	27,663.00 ± 0.6	467.04 ± 0.36
3	Syringin	12.27	265	373	9245.97 ± 0.54	74,535.30 ± 2.3	14,653.98 ± 0.5	1214.39 ± 0.89
4	**10**-Hydroxy-oleuropein	13.19	235	556	2153.98 ± 0.89	8943.89 ± 1.44	3405.45 ± 0.88	300.31 ± 1.25
5	Acteoside	13.95	324	625	5813.26 ± 1.02	2658.10 ± 0.88	9408.78 ± 0.59	-
6	Echinacoside	15.13	328	787	4537.26 ± 0.45	38,299.52 ± 0.1	7417.81 ± 1.23	2151.03 ± 0.71
7	Quercetin-rutinoside (Rutin)	15.78	256, 355	611	4779.29 ± 0.35	1604.54 ± 1.23	7642.07 ± 0.56	72.32 ± 0.99
8	Ligstroside	16.10	275	525	6921.53 ± 1.25	24,820.71 ± 2.0	11,019.22 ± 0.21	565.12 ± 3.01
9	Demethyl oleuropein	16.41	231	527	28,607.49 ± 0.71	6396.82 ± 0.55	35,729.89 ± 0.2	5345.42 ± 0.21
10	Oleuropein	17.12	280	540	4224.40 ± 0.89	9139.07 ± 0.78	6744.01 ± 0.58	-
11	Kaempferol-glucoside	17.42	265, 341	449	2377.10 ± 0.21	1690.59 ± 0.25	3814.93 ± 1.02	462.39 ± 0.66
12	Syringalactone A	17.96	223	511	8457.43 ± 0.65	17,161.82 ± 1.3	13,652.60 ± 0.6	1380.14 ± 1.22
13	Nuzhenide	18.33	275	687	9928.59 ± 1.02	129.65 ± 1.03	15,893.68 ± 0.3	1365.43 ± 0.5
14	Oleuropein-aglycone	18.85	280	379	7541.38 ± 1.56	42,796.39 ± 2.0	12,078.46 ± 0.7	1195.76 ± 0.25

Note: Values represent the mean ± standard deviations of three measurements.

**Table 2:** Peak positions (cm^−1^) and tentative assignments of FT-IR absorbance bands for *S. vulgaris* extracts from the leaves, bark, flowers and fruit recorded in the spectral region from 400 to 2000 cm^−1^.

*S. vulgaris* Leaves	*S. vulgaris* Bark	*S. vulgaris* Fruit	*S. vulgaris* Flowers	Tentative Assignement	References
~605	~605	~593	~598	β(CH)	[20,30]
~704	~704	~704	~705	γ(C=O)	[30]
~766	~766	~766	~774	C-O-C ring vibration	[29]
~816	~816	~816	~814	Phenyl-propanoid-associated signals of echinacoside	[21,22]
-	~853	~853	-	γ(CH)ar	[30]
~885	~890	~890	~884	-CH_2_ out-of-plane deformation (Terpene)	[23]
~930	~925	~925	~924	C-O-C ring vibration Symmetric stretching Rutin	[27,28,29]
~1026	~1033	~1032	~1039	-C-O, C-O-H, C-O-C, C-C	[20]
~1076	~1076	~1076	~1079	-C-OH stretching (iridoids skeletal vibration)	[24,25,26]
-	~1106	-	-	C-O stretch	[23,28]
1162	1162	1162	1159	C=C Stretching Rutin	[27,28]
~1268	~1261	~1256	~1263	C-O stretching (due to to terpenes, phenols or carbohydrates from iridoids)	[20,24,25]
~1404	~1385	~1394	~1401	symmetrical CH_3_ deformation Rutin	[20,23,27,28]
~1454	~1454	~1443	~1454	C-H asymmetric bending (esters, carbohydrate)	[25]
1515	1515	1515	~1516	Phenyl-propanoid-associated signals of echinacoside	[21,22]
1608	1608	1608	~1596	C=C stretching Rutin (iridoids skeletal vibration)	[24,26,27,28]
~1702	~1702	~1696	~1694	C=O stretch (terpenoids)	[23]

**Table 3:** Peak positions (cm^−1^) and tentative assignments of FT-IR absorbance bands for *Syringa vulgaris* extracts from the leaves, bark, flowers and fruit recorded in the spectral region from 2000 to 4000 cm^−1^.

*S. vulgaris* Leaves	*S. vulgaris* Bark	*S. vulgaris* Fruit	*S. vulgaris* Flowers	Tentative Assignement	References
2901	2901	2909		C-H stretch (terpenoid)	[23]
2933	2925	2933		-CH_2_ asymmetric stretching (from iridoids)	[20,24,25]
~3384	~3376	~3376		-OH stretching vibration of phenols, carboxylic acids and alcohols	[20,27,28]

**Table 4:** Total polyphenols content of the *S. vulgaris* extracts.

Sample	TPC (mg GAE/g)	TFC (mg RE/g)	TPA (mg CAE/g)
bark	3.36 ± 0.42	0.19 ± 0.24	3.99 ± 0.84
leaves	3.86 ± 0.31	0.71 ± 0.41	4.90 ± 0.42
flowers	3.98 ± 0.28	1.21 ± 0.12	2.36 ± 0.06
fruit	2.25 ± 0.02	0.75 ± 0.01	1.22 ± 0.01

Note: Values represent the mean ± SD of three independent measurements. TPC = total polyphenolic content; TFC = total flavonoids content; TPA = total phenolic acids content; GAE = gallic acid equivalents; RE = rutin equivalents; CAE = caffeic acid equivalents.

**Table 5:** The tentative identification of the main compounds in *S. vulgaris* flowers extract by GC-MS analysis.

Identified Compound	Retention Time (min)	Area	Content%
Benzyl alcohol	6.52	472,069	27.36 ± 0.19
Diethyl malonate	6.80	26,382	1.53 ± 1.29
5-ethoxydihydro-2-furanone	6.88	72,759	4.22 ± 0.95
3,4-dihydroxytetrahydro-2-furanone	7.43	86,244	5.00 ± 0.31
Lilac alcohol C	8.86	132,859	7.70 ± 1.02
Lilac alcohol D	8.98	569,808	33.02 ± 0.88
5-oxotetrahydrofuran-2 carboxylic acod, ethyl ester	9.11	33,140	1.92 ± 0.89
2-methyl-propanoic acid, propyl ester	9.45	34,760	2.01± 0.10
3-Phenylpropanal	10.27	23,867	1.83 ± 0.09
4-hydroxy-2-methylacetophenone	10.41	40,576	2.35 ± 0.92
Ethyl 3,3-diethoxypropionate	10.44	64,276	3.72 ± 0.37
2-hydroxy-3-methylsuccinic acid	10.51	16,343	0.95 ± 0.41
Tetrahydro [2,2] bifuranyl-5-one	10.59	10,896	0.63 ± 0.56
4-hydroxy-benzaldehyde	10.81	23,664	1.50 ± 1.16
Methyleugenol	11.50	14,156	0.82 ± 0.72
p-hydroxycinnamic acid, ethyl ester	15.39	15,797	0.92 ± 0.19
Tetradecanoic acid, ethyl ester	16.33	31,256	1.81 ± 1.02
n-hexadecanoic acid	18.66	38,934	2.26 ± 0.94
11,14-eicosadienoic acid, methyl ester	21.66	7722	0.45 ± 0.01

Note: Values represent the mean ± SD of three measurements.

**Table 6:** The tentative identification of the main compounds in *S. vulgaris* bark extract by GC-MS analysis.

Identified Compound	Retention Time (min)	Area	Content%
á-Psi-Carotene	6.50	663.200	0.61 ± 0.22
Benzoic acid, 4 formyl, methyl ester	11.14	13,207.20	12.20 ± 0.79
2-metoxyphenol	15.81	5445.60	5.03 ± 0.64
n-Hexadecanoic acid	18.66	8976.40	8.35 ± 0.09
Astaxanthin	19.31	1347.60	1.24 ± 0.12
trans-Sinapyl alcohol	19.42	15,466.00	14.28 ± 0.90
Oleic acid	21.77	17,973.60	16.06 ± 0.02

Note: Values represent the mean ± SD of three measurements.

**Table 7:** The tentative identification of the main compounds in *S. vulgaris* leaves extract by GC-MS analysis.

Identified Compound	Retention Time (min)	Area	Content%
Benzyl alcohol	6.54	4403.200	3.26 ± 0.75
Benzofuran,2,3-dihydro	8.92	5456.000	4.05 ± 0.95
Benzoic acid, 4-formyl, methyl ester	11.15	10,302.400	7.64 ± 0.01
Benzaldehyde, 2-hydroxy-6-methyl	11.94	12,287.600	9.12 ± 0.56
á-Phellandrene	13.15	1151.200	0.85 ± 0.22
4-{(1E)-3-Hydroxy-1-propenyl)-2-metoxyphenol	15.82	5185.200	3.85 ± 0.33
n-Hexadecanoic acid	16.67	5146.400	3.83 ± 0.57

Note: Values represent the mean ± SD of three measurements.

**Table 8:** The tentative identification of the main compounds in *S. vulgaris* fruit extract by GC-MS analysis.

Identified Compound	Retention Time (min)	Area	Content%
n-Hexadecanoic acid	18.67	17,880.400	11.83 ± 0.06
trans-13-octadecenoic acid	21.60	6479.200	4.28 ± 0.09
Trans-13-octadecanoic acid	21.77	32,274.00	21.36 ± 0.22
Oleic acid	21.85	8079.60	5.35 ± 0.25
Octadecanoic acid	22.18	12,766.80	8.45 ± 0.36

Note: Values represent the mean ± SD of three measurements.

**Table 9:** Antioxidant activity of *S. vulgaris* extracts by different assays.

Sample	DPPH (IC_50_ µg/mL)	FRAP (µM TE/g)	CUPRAC (µM TE/g)
bark	956 ± 1.71	157.92 ± 1.74	279.4 ± 1.18
leaves	865 ± 1.10	178.92 ± 0.62	169.7 ± 0.73
flowers	36.83 ± 0.47 *	182.52 ± 0.99 *	329.3 ± 0.15 *
fruit	103.19 ± 1.02	116.85 ± 0.42	110.5 ± 1.04

Note: Values represent the mean ± SD of three independent measurements. * *p* < 0.001.

**Table 10:** In vitro antiproliferative activity of *S. vulgaris* ethanolic extracts expressed as half maximal inhibitory concentration (IC_50_) (µmol GAE/mL) against B16F10 cells and HeLa cell lines.

IC_50_	B16F10	HeLa
Flower	5.74 ± 0.20	5.13 ± 0.12
Leaves	3.08 ± 0.23	4.91 ± 0.25
Fruit	2.28 ± 0.6	2.74 ± 0.4
Bark	2.62 ± 0.09	3.32 ± 0.09
Standard: Cisplatin 0.2 μM/mL		

Note: Values represent the mean ± SD of three independent measurements.

**Table 11:** GC-MS temperature gradient.

Time	Temperature	Rate
0 min	80 °C	0 °C/min
7 min	220 °C	20 °C/min
11 min	240 °C	5 °C/min
24 min	240 °C	0 °C/min

## Data Availability

Data sharing not applicable.

## Sample Availability

Samples of the compounds are not available from the authors.
